# Supplementary material for: Photophysical Deactivation Mechanisms of the Pyrimidine Analogue 1-Cyclohexyluracil
Source: Molecules. 2021 Aug 27;26(17):5191. doi: 10.3390/molecules26175191 (PMC8434193; doi:10.3390/molecules26175191)
Supplement: Supplementary file 1 [file molecules-26-05191-s001.zip › supporting-information.pdf]

# Photophysical Deactivation Mechanisms of the Pyrimidine Analogue 1-Cyclohexyuracil

Danillo Valverde*, Adalberto V. S. de Araújo and Antonio Carlos Borin*
Department of Fundamental Chemistry, Institute of Chemistry, University of São Paulo, Avenida Professor Lineu Prestes, 748, São Paulo, SP, CEP 05508-000, Brazil
*Email: dpvalverde@usp.br, ancborin@iq.usp.br

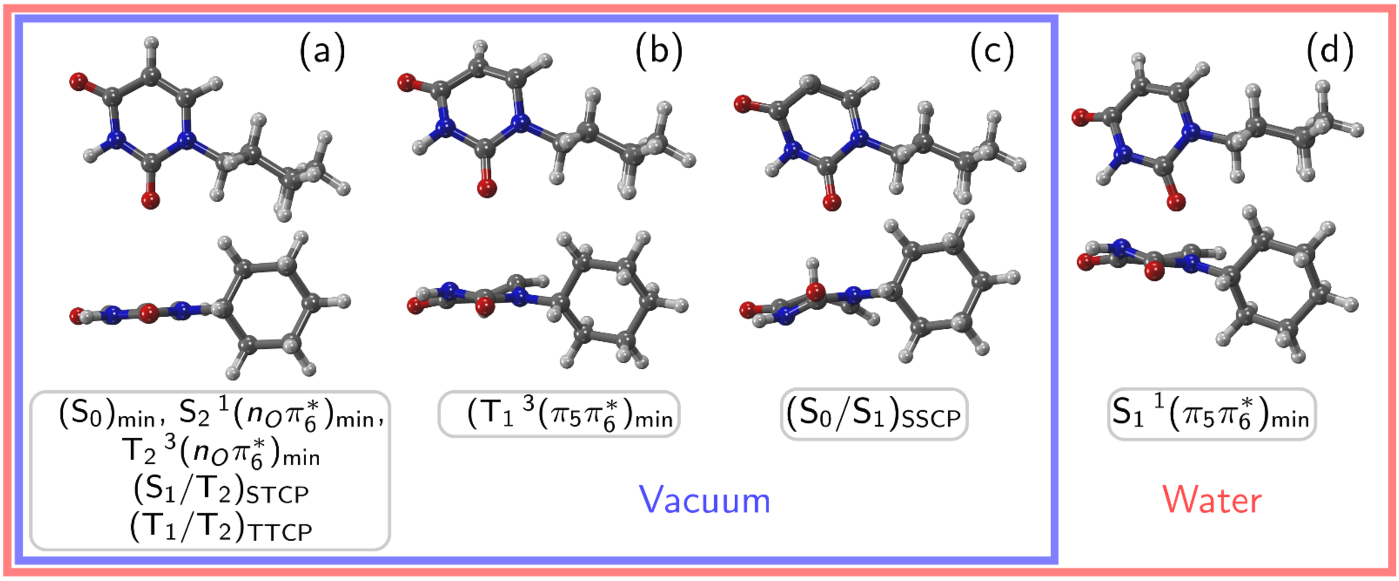


**Figure S1:** Critical point structures of 1-cyclohexyluracil in vacuum (blue line) and water (red line) optimized at the MS-CASPT2(12,9)/cc-pVDZ level of theory. Multiple labels below the geometry indicate that the geometries are similar.

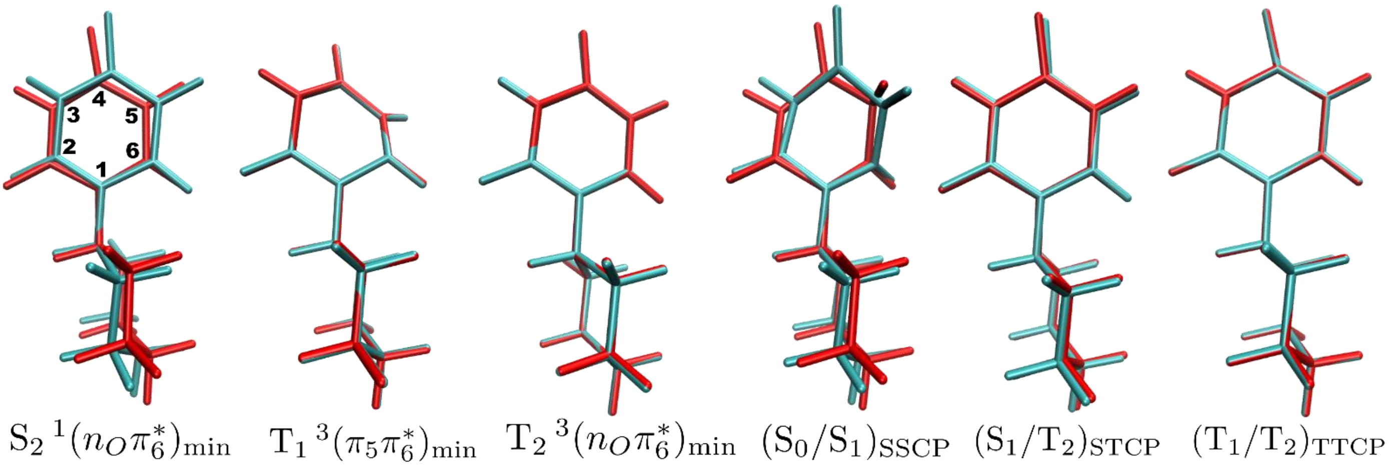


**Figure S2:** Superposition of the optimized structures obtained in gas phase (red) and water (cyan).

**Table S1**: The two low-lying singlet excited states for uracil, 1-cyclohexyluracil (1CHU), 1-trifluoromethyluracil (1CF3U), and 1-methoxyuracil (1OMEU) at the Franck-Condon region. Both optimization of the ground state as the vertical excitation energies calculation were obtained at the MS(3)-CASPT2(12,9)/cc-pVDZ level.

| Nature | Uracil | 1CHU | 1CF3U | 1OMEU |
|---|---|---|---|---|
| $^1(n\pi^*)$ | 4.87 (0.000) | 4.84 (0.001) | 4.79 (0.001) | 4.85 (0.001) |
| $^1(\pi\pi^*)$ | 4.98 (0.285) | 4.71 (0.360) | 5.11 (0.256) | 4.71 (0.303) |

**Table S2**: Influence of the atomic basis sets and active spaces on the nature and energetic order of the electronic states in vacuum and in water (oscillator strength in parenthesis). PCM method is used to take into account the effects of water.

| | cc-pVDZ | | | cc-pVTZ | | |
|---|---|---|---|---|---|---|
| | MS-CASPT2(12,9) | | MS-CASPT2(14,10) | MS-CASPT2(12,9) | | MS-CASPT2(14,10) |
| State | 4S+3T | 5S+4T | 6S+5T | 4S+3T | 5S+4T | 6S+5T |
| | | | Vaccuum | | | |
| $T_1$ | 3.73 | 3.57 | 3.65 | 3.66 | 3.57 | 3.57 |
| $S_1$ | 4.67(0.31) | 4.39(0.27) | 4.63(0.25) | 4.45(0.30) | 4.39(0.27) | 4.41(0.24) |
| $T_2$ | 4.73 | 4.63 | 4.68 | 4.68 | 4.63 | 4.53 |
| $S_2$ | 4.83(0.00) | 4.65(0.00) | 4.77(0.00) | 4.75(0.00) | 4.75(0.00) | 4.69(0.00) |
| $T_3$ | 5.46 | 5.01 | 5.09 | 5.40 | 5.01 | 4.97 |
| $S_3$ | 5.92(0.03) | 5.89(0.02) | 5.92(0.02) | 5.75(0.00) | 5.89(0.02) | 5.74(0.02) |
| $T_4$ | — | 5.97 | 6.07 | — | 5.97 | 5.97 |
| $S_4$ | — | 6.71(0.00) | 6.13(0.00) | — | 6.71(0.00) | 5.98(0.00) |
| | | | Water | | | |
| $T_1$ | 3.81 | 3.71 | 3.70 | 3.76 | 3.64 | 3.63 |
| $S_1$ | 4.63(0.316) | 4.59(0.269) | 4.59(0.252) | 4.41(0.315) | 4.36(0.268) | 4.37(0.248) |
| $T_2$ | 4.90 | 4.86 | 4.87 | 4.90 | 4.85 | 4.85 |
| $S_2$ | 5.04(0.001) | 5.02(0.000) | 4.99(0.001) | 5.00(0.000) | 4.97(0.000) | 4.95(0.000) |
| $T_3$ | 5.55 | 5.08 | 5.09 | 5.53 | 4.99 | 4.99 |
| $S_3$ | 6.04(0.036) | 6.07(0.021) | 6.03(0.021) | 5.89(0.038) | 5.95(0.021) | 5.87(0.020) |
| $T_4$ | — | 6.15 | 6.12 | — | 6.01 | 6.05 |
| $S_4$ | — | 6.97(0.000) | 6.27(0.000) | — | 6.83(0.000) | 6.15(0.000) |

## Molecular Geometries

On the following pages, all geometries employed for calculations in this work are given in XYZ file format

```
28
(S0)min in gas phase
N  -0.00934476   0.05434883  -0.01056622
C  -0.11079013   0.39557728   1.32602837
C  -1.27836859   0.34795616   2.02973617
C  -2.50255628  -0.09130989   1.36867902
N  -2.29914085  -0.40295119   0.00939356
C  -1.13360831  -0.36756331  -0.73965604
O  -3.61468918  -0.19763015   1.87252594
O  -1.09092114  -0.67733738  -1.92341761
H   0.82527811   0.71077571   1.79306723
H  -1.31976795   0.62117564   3.08405855
H  -3.12480832  -0.71188398  -0.50332050
C   1.28508153   0.12604982  -0.71422185
C   1.82154850   1.56094842  -0.76402071
C   3.13697046   1.60704059  -1.55419823
C   4.17164731   0.64332675  -0.95975638
C   3.62103932  -0.78730880  -0.90710015
C   2.30793188  -0.84473668  -0.11378346
H   1.05128289  -0.19843354  -1.74041999
H   2.00314263   1.93047463   0.26419466
H   1.06262532   2.22073256  -1.22030287
H   2.50963285  -0.57381043   0.94104096
H   1.88905621  -1.86650462  -0.11555525
H   3.52955793   2.63858549  -1.57027123
H   2.93630299   1.32407720  -2.60525740
H   4.36084771  -1.47317877  -0.45906901
H   3.43545214  -1.14506824  -1.93793800
H   5.10508404   0.67289449  -1.54856431
H   4.42969907   0.97269856   0.06590564
```

```
28
(T1)min in gas phase
N  -0.01352336   0.05381711  -0.00951249
C  -0.26875499   0.36479988   1.32633742
H   0.58826003   0.72078508   1.90092934
C  -1.60509640   1.00659923   1.48998815
H  -1.75720940   1.95161392   2.01922854
C  -2.71954245   0.43958555   0.75929056
O  -3.90468259   0.71292603   0.89972130
N  -2.29538580  -0.51529751  -0.18850282
H  -3.03213082  -0.87070749  -0.79875754
C  -1.03190820  -0.50877550  -0.79259562
O  -0.84191576  -0.97856481  -1.90733579
C   1.35956208   0.01904525  -0.54791407
C   2.02135874   1.39901215  -0.47355780
C   2.20419737  -1.05484823   0.15029417
H   1.24101173  -0.26357048  -1.60607478
C   3.42609114   1.34109185  -1.08999946
H   2.10227115   1.71860240   0.58243799
H   1.38849765   2.13949042  -0.99392652
C   3.61114072  -1.10022000  -0.46184243
H   2.27710492  -0.82329851   1.22982840
H   1.70073781  -2.03300611   0.05680488
C   4.28647797   0.27566759  -0.39858071
H   3.90622106   2.33280657  -1.02235982
H   3.34044440   1.09917788  -2.16679239
H   4.22251567  -1.85771896   0.05869466
H   3.53516928  -1.42079426  -1.51864929
H   5.28766842   0.23266635  -0.86181264
H   4.43591126   0.56013962   0.66141805
```

```
28
(S2)min in gas phase
N  -0.01020960   0.05423878  -0.01034813
C  -0.11187014   0.40230849   1.35172835
C  -1.35760195   0.30740425   2.01276046
C  -2.44458342  -0.11634702   1.28610142
N  -2.30380941  -0.43192025  -0.06706811
C  -1.09091659  -0.37668646  -0.76339219
O  -3.71510671  -0.27843070   1.72563300
O  -1.03865225  -0.69186995  -1.94769547
H   0.80338609   0.72600878   1.84186837
H  -1.45710363   0.56231669   3.06749585
H  -3.09527025  -0.76142500  -0.61183623
C   1.29253261   0.14125873  -0.68843892
C   1.81829458   1.58176327  -0.72075285
C   3.14631480   1.64427927  -1.48790624
C   4.18032302   0.68894039  -0.87900899
C   3.64255575  -0.74704763  -0.84216955
C   2.31662792  -0.82009979  -0.07244450
H   1.09091141  -0.17962857  -1.72238667
H   1.97586064   1.94739860   0.31149466
H   1.06106424   2.23637078  -1.18706878
H   2.49399834  -0.55166670   0.98610164
H   1.90799104  -1.84586271  -0.08511642
H   3.52982350   2.67953361  -1.49424540
H   2.96842957   1.36230993  -2.54346403
H   4.38206919  -1.42758035  -0.38513092
H   3.47980644  -1.10072855  -1.87841181
H   5.12446501   0.73066810  -1.44993589
H   4.41519162   1.01628006   0.15265415
```

```
28
(T2)min in gas phase
N  -0.01697960   0.05337731  -0.00864093
C  -0.06599301   0.41254085   1.35642821
C  -1.30448797   0.59264967   2.00175722
C  -2.45302690   0.37219453   1.27077305
N  -2.36328218  -0.02957768  -0.06295841
C  -1.15831307  -0.14280281  -0.77095980
O  -3.71943993   0.49204267   1.70673798
O  -1.16708927  -0.41239305  -1.96373228
H   0.89284185   0.57551984   1.84243966
H  -1.36600123   0.87127382   3.05258159
H  -3.20254830  -0.14211903  -0.62421618
C   1.28922657  -0.03856957  -0.68370790
C   2.01931588   1.31081537  -0.67070790
C   3.33867976   1.21879499  -1.44745622
C   4.23460612   0.11476357  -0.87389674
C   3.50248744  -1.23362567  -0.86228381
C   2.17125374  -1.15083293  -0.09769882
H   1.04611391  -0.29722497  -1.72563436
H   2.23874208   1.60929691   0.37173400
H   1.35741351   2.08344919  -1.09771910
H   2.37669420  -0.94902848   0.97047243
H   1.63658414  -2.11555322  -0.14587899
H   3.85954296   2.19190771  -1.42549973
H   3.12519313   0.99691143  -2.51040045
H   4.14724633  -2.01273061  -0.41929204
H   3.30252890  -1.54623365  -1.90508326
H   5.16923737   0.03711342  -1.45721931
H   4.53046382   0.38051530   0.15984874
```

```
28
 (S0/S1)SSCP in gas phase
N   0.01201458   0.04371155  -0.00969558
C  -0.37041422   0.32386265   1.25802064
C  -1.64121155  -0.24997603   1.72546145
C  -2.78148682   0.03036963   0.82869809
N  -2.30583075   0.33982338  -0.49810590
C  -1.05891553  -0.01702294  -0.99000370
O  -3.98150502  -0.09782129   1.01214739
O  -0.81673896  -0.23133577  -2.15939959
H   0.28429386   0.97266528   1.85419648
H  -1.55350591  -1.35747948   1.76457863
H  -3.02883444   0.35363473  -1.21625218
C   1.39835275   0.13657624  -0.52016186
C   2.07317929   1.45930370  -0.14192476
C   3.48795425   1.50422893  -0.74004927
C   4.32676413   0.30880086  -0.26823437
C   3.64021280  -1.01559899  -0.61938200
C   2.21515713  -1.07320014  -0.05024274
H   1.29143902   0.09104299  -1.61439743
H   2.15084959   1.55559131   0.95588948
H   1.46571044   2.30615314  -0.50446448
H   2.24587830  -1.07751900   1.05722208
H   1.70070920  -1.99955944  -0.36375801
H   3.97760347   2.45698094  -0.46856297
H   3.41109836   1.48613726  -1.84408964
H   4.22039751  -1.87824773  -0.24702808
H   3.58406218  -1.11600269  -1.71926262
H   5.33569751   0.34802721  -0.71460005
H   4.45869373   0.37306293   0.82830229
```

```
28
 (S1/T2)STCP in gas phase
N  -0.01020960   0.05423878  -0.01034813
C  -0.11187014   0.40230849   1.35172835
C  -1.35760195   0.30740425   2.01276046
C  -2.44458342  -0.11634702   1.28610142
N  -2.30380941  -0.43192025  -0.06706811
C  -1.09091659  -0.37668646  -0.76339219
O  -3.71510671  -0.27843070   1.72563300
O  -1.03865225  -0.69186995  -1.94769547
H   0.80338609   0.72600878   1.84186837
H  -1.45710363   0.56231669   3.06749585
H  -3.09527025  -0.76142500  -0.61183623
C   1.29253261   0.14125873  -0.68843892
C   1.81829458   1.58176327  -0.72075285
C   3.14631480   1.64427927  -1.48790624
C   4.18032302   0.68894039  -0.87900899
C   3.64255575  -0.74704763  -0.84216955
C   2.31662792  -0.82009979  -0.07244450
H   1.09091141  -0.17962857  -1.72238667
H   1.97586064   1.94739860   0.31149466
H   1.06106424   2.23637078  -1.18706878
H   2.49399834  -0.55166670   0.98610164
H   1.90799104  -1.84586271  -0.08511642
H   3.52982350   2.67953361  -1.49424540
H   2.96842957   1.36230993  -2.54346403
H   4.38206919  -1.42758035  -0.38513092
H   3.47980644  -1.10072855  -1.87841181
H   5.12446501   0.73066810  -1.44993589
H   4.41519162   1.01628006   0.15265415
```

```
28
 (T1/T2)TTCP in gas phase
N  -0.00993243   0.05292876  -0.00973904
C  -0.11007561   0.40832948   1.37397931
C  -1.29093878   0.35033662   2.05877512
C  -2.39266987  -0.06851628   1.34015963
N  -2.32194932  -0.39160027  -0.02214432
C  -1.09595538  -0.36287891  -0.72904015
O  -3.72017206  -0.27606005   1.67237027
O  -1.10379889  -0.69194456  -1.90907342
H   0.81735765   0.72928300   1.84480615
H  -1.34692778   0.61653764   3.11292163
H  -3.09703585  -0.74232170  -0.57669999
C   1.28475673   0.12949251  -0.69415766
C   1.82393482   1.56497075  -0.74210013
C   3.14354346   1.61683950  -1.52452420
C   4.17925608   0.65717523  -0.92540822
C   3.63151705  -0.77453460  -0.87309784
C   2.31350127  -0.83393337  -0.08888116
H   1.07403985  -0.19433333  -1.72621290
H   1.99692565   1.93620503   0.28647320
H   1.06589888   2.22350440  -1.20194631
H   2.50642833  -0.56252901   0.96696669
H   1.89816526  -1.85725208  -0.09060253
H   3.53514966   2.64909999  -1.54026504
H   2.95144803   1.33192230  -2.57705562
H   4.37275350  -1.45696898  -0.42147863
H   3.45433533  -1.13295414  -1.90557843
H   5.11611513   0.68909002  -1.50963970
H   4.43133953   0.98829416   0.10121145
```

```
28
 (S0)min in water
N  -0.01271902   0.03845108  -0.01874283
C  -0.09555727   0.37794962   1.31488917
C  -1.26399006   0.34823425   2.03029241
C  -2.49444991  -0.04315007   1.36576687
O  -3.62191393  -0.09338130   1.86995565
N  -2.30703821  -0.38996647   0.01958340
C  -1.14610111  -0.35811269  -0.73417359
O  -1.12695662  -0.65544554  -1.92726409
C   1.28480844   0.10381870  -0.72510463
C   1.82394425   1.53813339  -0.77227296
C   2.30324292  -0.86710977  -0.11663572
C   3.14668699   1.58346789  -1.55017349
C   3.62372684  -0.81061184  -0.89763927
C   4.17556182   0.61971495  -0.94579642
H   0.84798694   0.68527479   1.77633829
H  -1.28720032   0.62905784   3.08607673
H  -3.15151163  -0.66832765  -0.50066886
H   1.06242215  -0.22096966  -1.75413580
H   1.99620562   1.90841375   0.25705424
H   1.06946266   2.19793109  -1.23599323
H   2.49541992  -0.59625848   0.93976737
H   1.88381860  -1.88870990  -0.12199909
H   3.53999797   2.61489772  -1.56336174
H   2.95594008   1.29994893  -2.60304917
H   4.35928885  -1.49670783  -0.44273678
H   3.44777329  -1.16854930  -1.93022598
H   5.11456016   0.64868907  -1.52593347
H   4.42420459   0.94942142   0.08209097
```

```
28
  (S1)min in water
N   0.02292489  -0.09508096   0.07762557
C   0.00179932   0.11701833   1.41447522
C  -1.32017270   0.58604224   1.93481336
C  -2.50996178   0.15219104   1.30593988
O  -3.67140628   0.38054640   1.57235012
N  -2.24304816  -0.69842492   0.12501104
C  -1.14371272  -0.53129138  -0.66364766
O  -1.05649475  -0.75798039  -1.87737707
C   1.28216564   0.05099218  -0.68418476
C   1.78476799   1.49847238  -0.63573123
C   2.34503442  -0.93719364  -0.19027811
C   3.07680887   1.64013838  -1.45267272
C   3.63443970  -0.78421532  -1.00934573
C   4.14978815   0.65970521  -0.96262621
H   0.96801908   0.34046044   1.88442839
H  -1.33643177   1.32487949   2.75445589
H  -3.09525795  -0.95435163  -0.39547170
H   1.03025798  -0.19772940  -1.72751077
H   1.98553773   1.79117617   0.41322742
H   0.99831849   2.17227542  -1.01926304
H   2.56906074  -0.74517501   0.87706352
H   1.95024566  -1.96605463  -0.26239076
H   3.44459703   2.67955452  -1.39736680
H   2.85464092   1.43525136  -2.51755629
H   4.40229492  -1.48404099  -0.63603500
H   3.42957072  -1.06446084  -2.06044150
H   5.06604976   0.75926988  -1.57062796
H   4.42778209   0.91413068   0.07904091
```

```
28
  (S2)min in water
N   0.90587983   0.82405983  -0.05063834
C   0.15075296   2.01713605  -0.07348924
C   0.81554156   3.26122017  -0.00150712
C   2.18918084   3.25883693   0.01452958
O   2.99886517   4.34925817   0.05881147
N   2.90157548   2.06098714  -0.09585745
C   2.28551375   0.81002028  -0.00364436
O   2.97238246  -0.21100536   0.08183834
C   0.19896396  -0.47077702  -0.03010239
C  -0.62254189  -0.63720138   1.25347510
C  -0.66221916  -0.65554812  -1.28483040
C  -1.31342699  -2.00807236   1.26830067
C  -1.35286619  -2.02637129  -1.25819959
C  -2.18306376  -2.20125705   0.01964520
H  -0.93621585   1.91099028  -0.04549742
H   0.25702741   4.20006571   0.03459379
H   3.90441024   2.05473960   0.13168860
H   0.99610755  -1.23128640  -0.03704352
H  -1.39210833   0.15690946   1.31221048
H   0.03894815  -0.51803904   2.12974791
H  -1.43349404   0.13772956  -1.33098708
H  -0.02847173  -0.54919493  -2.18297433
H  -1.91932756  -2.11391416   2.18504975
H  -0.54150457  -2.80077468   1.30130015
H  -1.98702507  -2.14537460  -2.15395628
H  -0.58213864  -2.81962351  -1.30377544
H  -2.65836471  -3.19773065   0.03428549
H  -3.00238088  -1.45578260   0.02702642
```

```
28
  (T1)min in water
N  -0.01673874   0.03365518  -0.01962854
C  -0.26003368   0.34756095   1.32043927
C  -1.59158337   1.01026532   1.48732021
C  -2.70140557   0.45329029   0.75680889
O  -3.88760848   0.73518345   0.92346319
N  -2.30570998  -0.49514395  -0.20176627
C  -1.03683888  -0.51594344  -0.79447556
O  -0.85135728  -1.00045522  -1.90758374
C   1.35798774   0.00654044  -0.56111734
C   2.00667230   1.39255931  -0.48319569
C   2.20658857  -1.05868970   0.14565870
C   3.41684819   1.34654169  -1.08817965
C   3.61886163  -1.09221902  -0.45492462
C   4.28125325   0.28985131  -0.38828394
H   0.60520064   0.71219544   1.88434992
H  -1.72623407   1.94786313   2.05267589
H  -3.05513166  -0.86233044  -0.80587036
H   1.25086769  -0.27857130  -1.61982923
H   2.07627437   1.71408158   0.57302125
H   1.37136346   2.12670098  -1.00960035
H   2.26865490  -0.82487683   1.22538594
H   1.71274833  -2.04152639   0.04963751
H   3.88745514   2.34267266  -1.01807465
H   3.34205976   1.10242157  -2.16528482
H   4.23281657  -1.84338769   0.07178927
H   3.55437061  -1.41500952  -1.51181860
H   5.28650280   0.25525428  -0.84340072
H   4.41964191   0.57711630   0.67246618
```

```
28
  (T2)min in water
N  -0.01935842   0.07215409  -0.02111147
C  -0.05444682   0.40959240   1.35251920
C  -1.29003152   0.56169678   2.00746630
C  -2.44204858   0.34831417   1.27494374
O  -3.70770156   0.45271850   1.73810768
N  -2.36339064  -0.04904757  -0.06398393
C  -1.16381048  -0.13581256  -0.76848758
O  -1.16882758  -0.39965447  -1.97340280
C   1.28360207  -0.02397465  -0.70008936
C   2.02019695   1.32191793  -0.69374413
C   2.15426517  -1.13436572  -0.09388328
C   3.34671847   1.21545676  -1.45632750
C   3.49258097  -1.23153133  -0.84428161
C   4.23144394   0.11310066  -0.86251627
H   0.90954684   0.54548651   1.84673940
H  -1.33999376   0.84363191   3.06284529
H  -3.21493972  -0.09868060  -0.63729325
H   1.04954477  -0.29223556  -1.74166048
H   2.23076125   1.63012247   0.34769566
H   1.36638921   2.09328295  -1.13527304
H   2.35011126  -0.92249746   0.97414144
H   1.61533051  -2.09693074  -0.13742821
H   3.87214888   2.18620874  -1.43922757
H   3.14267558   0.98360389  -2.51899867
H   4.12905596  -2.00911831  -0.38687772
H   3.30141672  -1.55395942  -1.88573934
H   5.17141159   0.02487864  -1.43567247
H   4.51835922   0.38811687   0.17132665
```

```
28
  (S0/S1)SSCP in water
N   0.02863900  -0.06971900   0.04708200
C  -0.18768100   0.64391800   1.22591100
C  -1.15785600  -0.04566700   2.08775100
C  -2.45841700  -0.24349300   1.45507300
O  -3.52405400  -0.60159600   1.98270700
N  -2.34552100  -0.13173600   0.05568800
C  -1.15471000  -0.39561000  -0.64968100
O  -1.17144800  -0.81705300  -1.79874900
C   1.30562600   0.00516400  -0.69573000
C   1.78437100   1.45157200  -0.85935300
C   2.35648500  -0.86595800   0.00312400
C   3.10401700   1.47049900  -1.64645900
C   3.68701700  -0.82421900  -0.76235800
C   4.17424000   0.61616800  -0.95363900
H   0.07736900   1.70799000   1.28878800
H  -0.82108200  -0.88781200   2.73077600
H  -3.19222900  -0.37907300  -0.47910100
H   1.09795600  -0.41633800  -1.69075000
H   1.95521600   1.91802000   0.12742800
H   1.01037000   2.04567400  -1.37469400
H   2.49909500  -0.49615000   1.03769900
H   1.97527000  -1.90041200   0.07842100
H   3.45225700   2.51246800  -1.76574000
H   2.92026100   1.07512600  -2.66383500
H   4.43789400  -1.43321500  -0.22875800
H   3.53775400  -1.29250400  -1.75301600
H   5.11213100   0.62976200  -1.53579300
H   4.40353000   1.05904500   0.03389000
```

```
28
  (T1/T2)TTCP in water
N  -0.01002379   0.05306445  -0.00999308
C  -0.10946650   0.40815692   1.37336616
C  -1.29177014   0.34999493   2.05791822
C  -2.39395346  -0.06879184   1.33992868
O  -3.71994569  -0.27454216   1.67610641
N  -2.32158066  -0.39174541  -0.02219480
C  -1.09631994  -0.36257995  -0.72961480
O  -1.10328653  -0.69223788  -1.90968907
C   1.28481540   0.12958215  -0.69441466
C   1.82402520   1.56505125  -0.74227397
C   2.31344434  -0.83385846  -0.08896515
C   3.14376343   1.61689506  -1.52448109
C   3.63159013  -0.77448461  -0.87296517
C   4.17936152   0.65721593  -0.92519098
H   0.81795504   0.72919831   1.84409836
H  -1.34794406   0.61537660   3.11235702
H  -3.09727744  -0.74286662  -0.57601310
H   1.07426297  -0.19424420  -1.72650330
H   1.99685294   1.93628665   0.28632637
H   1.06607586   2.22359570  -1.20224743
H   2.50620216  -0.56245320   0.96691338
H   1.89809163  -1.85717028  -0.09075090
H   3.53538934   2.64914899  -1.54016150
H   2.95183646   1.33197697  -2.57704300
H   4.37274093  -1.45692954  -0.42122136
H   3.45457235  -1.13290521  -1.90547352
H   5.11631722   0.68911290  -1.50926841
H   4.43128153   0.98833466   0.10146887
```

```
28
  (S1/T2)STCP in water
N   0.01037100   0.01818200   0.02352700
C  -0.07304100   0.37489500   1.39722600
C  -1.28931000   0.36776300   2.03948200
C  -2.48778000  -0.01716100   1.35962100
O  -3.69539200   0.01813100   1.80204500
N  -2.32040000  -0.41258200  -0.00850200
C  -1.12632400  -0.38436600  -0.69044900
O  -1.07234200  -0.69495300  -1.88601600
C   1.29259200   0.08811400  -0.71081700
C   1.82129300   1.52650200  -0.75135700
C   2.32702600  -0.88214700  -0.12887000
C   3.13107000   1.58898600  -1.54973900
C   3.63442000  -0.80850900  -0.93020200
C   4.17587700   0.62598400  -0.97206900
H   0.86104200   0.67255600   1.86414000
H  -1.37934900   0.67871800   3.06648300
H  -3.14214700  -0.69347900  -0.55065600
H   1.05603800  -0.22743000  -1.73956500
H   2.00754900   1.88714500   0.27895500
H   1.05509500   2.18595800  -1.19595700
H   2.53429000  -0.62100800   0.92712700
H   1.91435500  -1.90646500  -0.13827400
H   3.51726100   2.62315200  -1.55829800
H   2.92536300   1.31520900  -2.60237700
H   4.38172700  -1.49427800  -0.49435000
H   3.44432900  -1.15684900  -1.96356500
H   5.10524100   0.66741700  -1.56678100
H   4.43876100   0.94661900   0.05514500
```

```
28
  (S1)TS in water
N  -0.00697013   0.01452692   0.03256046
C  -0.04355062   0.35906307   1.35496265
C  -1.35344868   0.32165925   2.07479543
C  -2.41787765  -0.11268341   1.35981860
O  -3.69069451  -0.25931313   1.75102205
N  -2.27705073  -0.46290956   0.05449327
C  -1.10525298  -0.40095732  -0.67142668
O  -1.10064906  -0.70524193  -1.84875413
C   1.28621795   0.08707629  -0.69941705
C   1.81814869   1.52350773  -0.75452582
C   2.31942814  -0.87986170  -0.10931592
C   3.12260411   1.56924103  -1.56381430
C   3.62737139  -0.81931125  -0.91114457
C   4.16994382   0.61323354  -0.97829242
H   0.88264633   0.64083092   1.82406798
H  -1.43036682   0.61798529   3.10724776
H  -3.09720592  -0.77659628  -0.44626711
H   1.04256217  -0.23844138  -1.72280347
H   2.01760323   1.89495311   0.26832430
H   1.05371054   2.18360201  -1.20030715
H   2.51903009  -0.60776202   0.94560765
H   1.90230877  -1.90270749  -0.10914749
H   3.51035265   2.60296317  -1.59167265
H   2.90726089   1.27938764  -2.61016867
H   4.37111006  -1.50148386  -0.46338128
H   3.43421978  -1.18428003  -1.93772420
H   5.09584246   0.64464075  -1.57886287
H   4.43828100   0.95023061   0.04163363
```
